# Targeting Bacterial Cell Division with Benzodioxane–Benzamide FtsZ Inhibitors as a Novel Strategy to Fight Gram-Positive Ovococcal Pathogens

**DOI:** 10.3390/ijms26020714

**Published:** 2025-01-16

**Authors:** Berenice Furlan, Marta Sobrinos-Sanguino, Marcella Sammartino, Begoña Monterroso, Silvia Zorrilla, Alessia Lanzini, Lorenzo Suigo, Ermanno Valoti, Orietta Massidda, Valentina Straniero

**Affiliations:** 1 Dipartimento di Biologia Cellulare e Integrata, Università degli Studi di Trento, Via Sommarive, 9, 38123 Trento, Italy; berenice.furlan@unitn.it; 2 Centro de Investigaciones Biológicas Margarita Salas, Consejo Superior de Investigaciones Científicas, Ramiro de Maeztu 9, 28040 Madrid, Spain; marta.sobrinos@gmail.com (M.S.-S.); marcella.sammartino@libero.it (M.S.); silvia@cib.csic.es (S.Z.); 3 Dipartimento di Scienze Farmaceutiche, Università degli Studi di Milano, Via Luigi Mangiagalli, 25, 20133 Milan, Italy; alessia.lanzini@unimi.it (A.L.); lorenzo.suigo@uth.tmc.edu (L.S.); ermanno.valoti@unimi.it (E.V.); 4 Instituto de Química Física Blas Cabrera, Consejo Superior de Investigaciones Científicas, Serrano 119, 28006 Madrid, Spain; bmonterroso@iqf.csic.es; 5 Department of Microbiology and Molecular Genetics, McGovern Medical School, University of Texas, Houston, TX 77030, USA; 6 Centro Interdipartmentale di Scienze Mediche, Via Santa M. Maddalena, 1, 38122 Trento, Italy

**Keywords:** antimicrobial resistance, novel targets, bacterial cell division, FtsZ inhibitors, benzodioxane–benzamides, *Streptococcus pneumoniae*

## Abstract

The widespread emergence of antimicrobial resistance (AMR) is a serious threat to global public health and among Gram-positive cocci, *Streptococcus pneumoniae* constitutes a priority in the list of AMR-threatening pathogens. To counteract this fundamental problem, the bacterial cell division cycle and the crucial proteins involved in this process emerged as novel attractive targets. FtsZ is an essential cell division protein, and FtsZ inhibitors, especially the benzamide derivatives, have been exploited in the last decade. In this work, we identified, for the first time, some benzodioxane–benzamide inhibitors capable of targeting FtsZ in *Streptococcus pneumoniae*, in addition to their previously demonstrated activity against other bacteria. These promising benzamides, with minimal inhibitory concentrations (MICs) ranging from 25 to 80 µg/mL, demonstrated bactericidal activity against *S. pneumoniae*. This was evidenced by their ability to dramatically affect growth and viability, further supported by the morphological changes observed through microscopy. Moreover, the compounds were characterized in vitro, combining turbidity measurements and confocal imaging, and significant alteration of a GTP-induced FtsZ assembly was found, in line with our previous data from other microorganisms.

## 1. Introduction

Antimicrobial resistance (AMR) is a public health emergency that poses a serious threat to humans globally [1]. Bacterial resistance has been detected in all the antibiotics currently in clinical use, while only a few antibacterial drugs have been approved recently [2] or are in clinical development [3]. Indeed, one of the major problems regarding AMR is that, since the 1970s, the development of antibiotics has decreased dramatically, and most of the new compounds commercially available are analogs of the existing ones [4,5,6]. This makes the design and development of antibiotics with novel mechanisms of action a crucial strategy to combat AMR.

Bacterial cell division is an essential process, the mechanism of which is still not completely understood. Interest in this process is due to an intrinsic biological curiosity and also the possibility of exploiting cell division proteins as primary targets to develop novel broad-spectrum antibacterials [7,8]. Substantial advances have been made in elucidating cell division in the model rod-shaped organisms *Escherichia coli* and *Bacillus subtilis* [8,9,10,11,12,13], as well as in the non-conventional pathogenic models with different morphologies [14,15,16,17,18].

The proteins involved in this process localize to the midcell to form the division complex, or divisome [10,15,16], which can be interconnected with the cell elongation complex, or elongasome, that carries out peripheral growth [19].

The bacterial filamenting temperature-sensitive Z (FtsZ) cell division protein is an essential protein conserved in almost all bacteria. FtsZ assembles at the midcell to form a ring (Z-ring), a membrane-attached structure required to recruit the other divisome proteins and to complete cytokinesis. When bound to GTP, FtsZ monomers polymerize into single-stranded filaments, known to laterally associate into bundles under in vitro conditions, mimicking the crowded intracellular environment [20,21,22], which impart the dynamic properties to the Z-ring during division [23]. Inactivation or inhibition of FtsZ causes a cell division block and, eventually, cell death, making it a very attractive target [24]. The functional and structural characterization of FtsZ has allowed the identification of compounds that are able to inhibit its activity [25].

Currently, several FtsZ inhibitors, both from natural and synthetic sources, have been reported in the literature [26]. They bind FtsZ in two main sites: the GTP-binding site and the interdomain-binding site. The majority of the inhibitors bind FtsZ in the latter site, and the most exploited and potent class of compounds are structural derivatives of 2,6-difluorobenzamide. Scientific interest in this class of compounds was prompted by the results obtained with PC190723, a benzamide ether derivative that stabilizes the FtsZ polymers against *Staphylococcus aureus* [27]. Since then, PC190723 has been heavily modified in its structure to achieve more promising derivatives [28,29,30,31,32,33,34,35,36] as well as prodrugs, one of which, TXA-709, is currently in clinical development [37,38].

Despite the conserved and widespread nature of FtsZ and the antibacterial activity of these inhibitors against *S. aureus*, the majority of research groups have not succeeded in broadening their spectrum against other bacterial species, including other Gram-positive pathogens. Nevertheless, we recently reported that FtsZ inhibitors could also be effective against *Mycobacterium tuberculosis* [31] and *E. coli* [39].

Among Gram-positive cocci, *Streptococcus pneumoniae* represents a problem within the context of AMR infections and AMR-related deaths [40], and it has been included in the list of pathogens for which the discovery of new antibiotics is urgently needed [41]. *S. pneumoniae* has an ovococcal morphology, which imposes a need for coordination between the elongation and the division complexes different from that of the rod-shaped models. In this species, FtsZ and its partner protein FtsA are recruited simultaneously to the midcell to orchestrate both sidewall elongation and cell division [17,18], although the mechanism still needs to be clarified.

Starting from the crucial role of FtsZ also in *S. pneumoniae*, in the latest years, Panda’s research group has suggested Vitamin K3 as a potential anti-pneumococcal drug that targets FtsZ, by inducing conformational changes in the protein that increase GTP hydrolysis, thus destabilizing the FtsZ polymers [42]. The same group then moved to the identification of narrow-spectrum antibacterial compounds showing an imidazo [1,2-a]pyridine-3-carboxylate moiety [43]. The most promising candidate showed the ability to inhibit the proliferation of the bacteria by inhibiting FtsZ polymerization without causing membrane damage.

**Figure 1:**
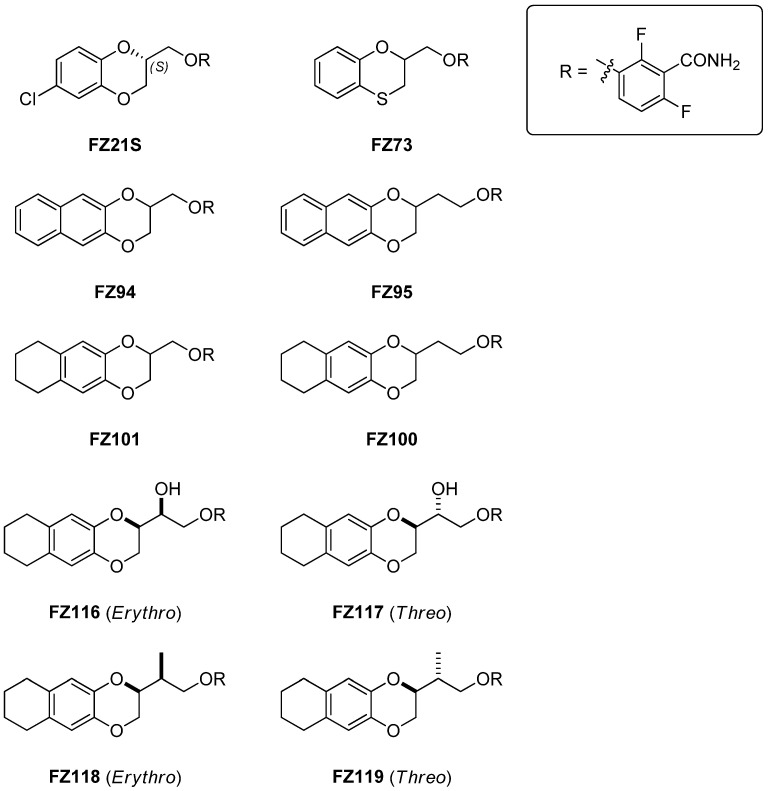
Benzodioxane–benzamides, object of the present work.

Considering this prior data and aiming to further broaden the spectrum of action of our benzodioxane–benzamides, we decided to test a selection of our most promising FtsZ inhibitors (see Figure 1) on *S. pneumoniae*. These candidates underwent a deep characterization and, with this work, for the first time, we identified and fully detailed four benzodioxane–benzamides (FZ95, FZ100, FZ116, and FZ118) as promising antibacterial FtsZ inhibitors towards ovoid-shaped Gram-positive pathogens. After a first evaluation of the antibacterial activities, we investigated if the compounds had a bactericidal effect by measuring cell growth and cell viability. Furthermore, we observed cell morphological defects consistent with a block at the early stages of *S. pneumoniae* cell division, similar to those observed upon FtsZ inactivation. In vitro turbidity assays and confocal microscopy analyses, conducted with purified *S. pneumoniae* FtsZ, further corroborated the postulated benzodioxane–benzamide mechanism of action. Our class of FtsZ inhibitors thus opens the possibility of developing broad-spectrum antimicrobials to be used over a variety of Gram-positive and Gram-negative strains.

## 2. Results

### 2.1. Chemistry

All compounds (FZ21S [30], FZ73 [33], FZ94 [35], FZ95 [35], FZ100 [35], FZ101 [35], FZ116 [44], FZ117 [44], FZ118 [36], and FZ119 [36]) were synthetized de novo following the methodologies reported in our prior works.

Subsequent NMR and HPLC analyses confirmed that each compound exhibited a purity exceeding 95% (see Supporting Information).

### 2.2. Antimicrobial Activity on S. pneumoniae

We firstly determined the minimal inhibitory concentrations (MICs) of the selected benzodioxane–benzamides against *S. pneumoniae*, using an agar dilution method [45]. Two reference wild-type strains were used for the assay: the clinical isolate D39 strain and the well-characterized laboratory derivative Rx1 strain. The results for the two strains are reported in Table 1 and are almost identical, with only a minimal difference observed for FZ116.

As clearly visible, four out of the ten tested benzodioxane–benzamide derivatives showed a promising MIC and underwent further studies to understand their effects on *S. pneumoniae* cells. Given the structural similarity between FtsZ and eukaryotic tubulin [46], the cytotoxicity of these compounds was already considered using the MTT assay on human MRC-5 cells, determining the doses (μg/mL) that reduce the viability of these cells by 90% (TD90), and all the selected benzodioxane–benzamides were considered safe towards eukaryotic cells.

### 2.3. Effect on Growth, Viability, and Morphology of S. pneumoniae Rx1 of Selected Benzodioxane–Benzamides

The promising MIC results on *S. pneumoniae*, along with the lack of eukaryotic cytotoxicity in MRC-5 cells, led us to perform growth and viability assays using concentrations of each compound that reflected their MIC. We determined the activity of the selected benzodioxane–benzamides against exponentially growing *S. pneumoniae* cells and evaluated the outcome to detect if they demonstrated a bacteriostatic, bactericidal, and/or bacteriolytic effect.

**Figure 2:**
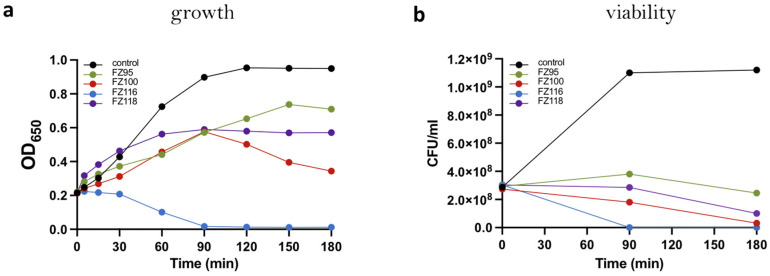
Effects of selected benzodioxane–benzamide compounds on growth (**a**) and viability (**b**) of *S. pneumoniae*. Cells were grown in TSB until 0.2 OD and then split into tubes in the absence (positive control) or in the presence of FZ95 and FZ100 (30 μg/mL), FZ116 (80 μg/mL), FZ118 (75 μg/mL) and incubated statically at 37 °C. ODs and viable counts were measured every 30 min and 90 min, respectively.

As shown in Figure 2, all compounds tested showed a clear bactericidal effect within 180 min of treatment. In the case of FZ116, this effect was also rapidly bacteriolytic, with a drop in OD and viable counts within 90 min.

**Figure 3:**
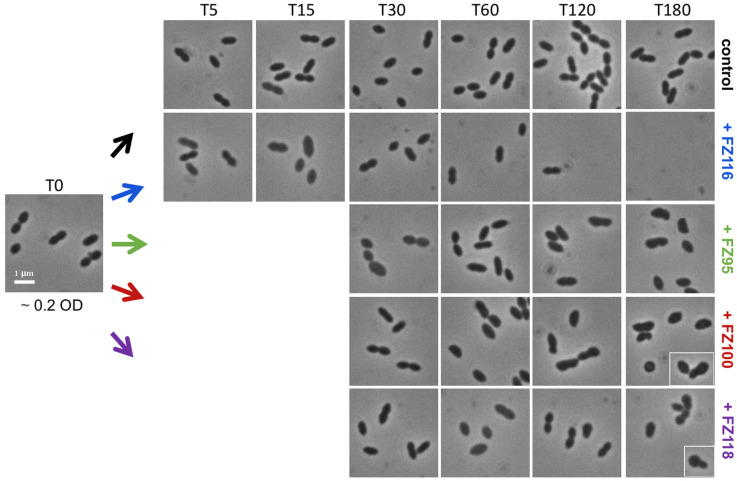
Effects of selected benzodioxane–benzamide compounds on the morphology of *S. pneumoniae.* Representative fields of cells at different times points were imaged using phase-contrast microscopy. The boxed sections in the images highlight significant morphological changes. Size bar, 1 μm.

Images by phase-contrast microscopy showed the morphological alterations that precede growth inhibition and death of *S. pneumoniae* cells treated with FZ95, FZ100, FZ116, and FZ118 benzamides at 1× MIC (Figure 3).

The effect eventually resulted in cell death and lysis with respect to the untreated control and is suggestive of a simultaneous block in sidewall elongation and division, as it would be expected from a block in the early stages of the pneumococcal cell cycle and consistent with the *S. pneumoniae* FtsZ depletion phenotype observed by Perez et al. [47]. The results in Figure 3 confirm what was observed with the growth and viability assay: the faster rate of killing was evident for FZ116, where the changes in morphology were already noted after 15 min from the addition of the compound. In all the other cases, cell enlargement and bulging could be observed only after 60 min and become clear after 120 to 180 min.

### 2.4. Effect of Benzodioxane–Benzamides on FtsZ Assembly in Crowding Conditions

**Figure 4:**
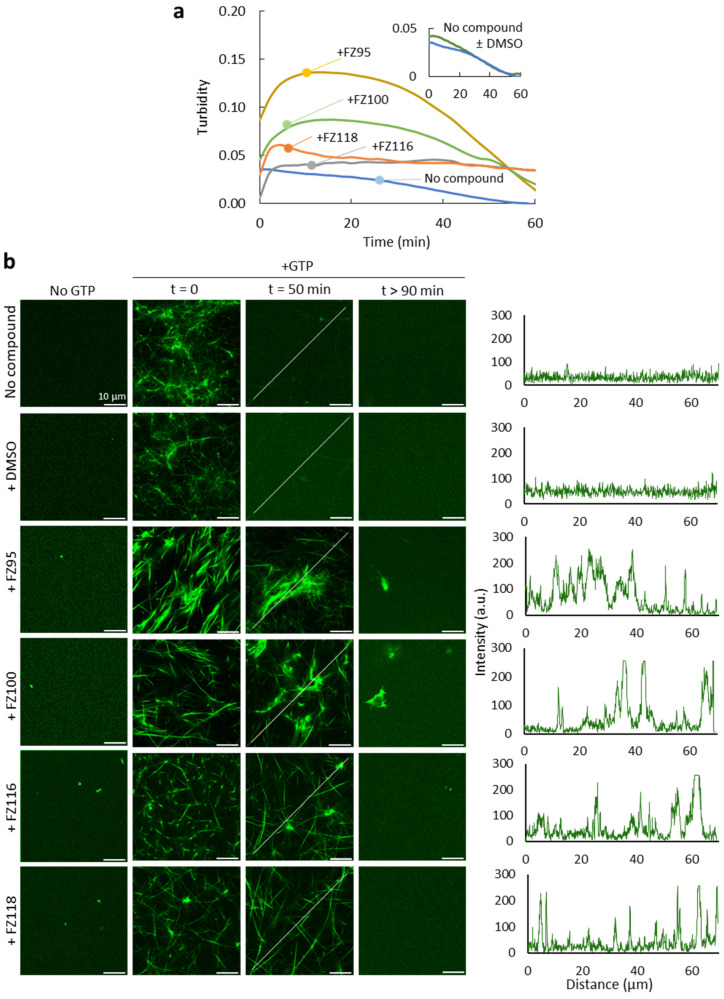
Effect of benzodioxane–benzamides on GTP-triggered FtsZ polymers in crowding conditions. (**a**) Representative depolymerization profiles of FtsZ in the absence and presence of the indicated compounds, obtained by turbidity at 350 nm. Inset shows depolymerization profiles of FtsZ with and without DMSO. (**b**) Representative confocal images of FtsZ before and after triggering polymerization with GTP, in the absence and presence of the stated compounds or DMSO, at the indicated times. Intensity profiles along the lines drawn in the images are shown on the right. FtsZ concentration was 12 µM, with 1 µM FtsZ-Alexa 488 in (**b**), compounds were at 20 µM and the buffer was 50 mM Tris-HCl, 100 mM KCl, 5 mM MgCl_2_, pH 7.5, with 150 g/L Ficoll 70. Polymerization was triggered by 0.5 mM GTP. Experiments performed in triplicates.

We next sought to determine the possible impact of the benzodioxane–benzamides displaying a clear killing activity on the GTP-triggered assembly of purified *S. pneumoniae* FtsZ under conditions mimicking the crowded nature of the bacterial cytoplasm. To this end, we conducted turbidity measurements showing the evolution of FtsZ polymers after induction by GTP in the absence and presence of FZ95, FZ100, FZ116, and FZ118 in solutions containing Ficoll 70 as a crowder (Figure 4a).

These measurements revealed significant changes in the presence of benzodioxane–benzamides. In the case of samples without any compound, turbidity values returned to basal levels around 50 min after the GTP addition, reflecting the time-dependent depolymerization due to GTP depletion. In the presence of the compounds, the polymer profiles were substantially modified. A marked increase in turbidity was observed in samples with FZ95 and FZ100. This result is compatible with the formation of larger polymeric structures and/or with a higher amount of assembled protein than in their absence. Full depolymerization, as detected by turbidity, seemed to take longer (>60 min) than in the absence of these compounds. The increase in the maximum turbidity reached was more modest in the case of FZ118 and almost negligible for the FZ116 compound. However, the longer lifetime of the polymers was more obvious in these cases, as no significant decrease in turbidity was observed in the time interval tested.

We also conducted confocal microscopy experiments under the same crowded conditions on samples containing FtsZ labeled with Alexa Fluor 488 (FtsZ-Alexa 488) as a tracer, with and without the compounds (Figure 4b). In all cases, we observed the formation of FtsZ bundles immediately after the GTP addition, as previously described for FtsZ from other species in solutions containing crowding agents [20,21,22]. Consistent with the turbidity measurements, polymers seemed somehow thicker and/or more clustered in the samples with FZ100 and, more so, with FZ95. We found a time-dependent depolymerization with and without the compounds, polymers fully disassembled at times longer than 90 min after the GTP addition, and with large structures occasionally remaining in the presence of FZ95 or FZ100. Interestingly, 50 min after the induction of polymerization, we clearly found polymers in the presence of all tested benzodioxane–benzamides, whereas in their absence, most of the protein was already unassembled.

Taken together, confocal and turbidity assays imply that compounds FZ95, FZ100, FZ116, and FZ118 hyperstabilize FtsZ bundles in crowding cytomimetic conditions, suggesting that this protein could be the target of the compounds in vivo.

## 3. Discussion

In this work, we aimed to further expand the spectrum of action of our benzodioxane–benzamide FtsZ inhibitors. These compounds initially demonstrated a strong ability to target the FtsZ proteins of some Gram-positive bacteria, specifically *S. aureus* and *B. subtilis* [34,36]. More recently, they have also been confirmed to be active against two *E. coli* strains that are defective in the AcrAB-TolC efflux system [39].

The present results revealed that our benzodioxane–benzamides also exhibit a very promising activity against *S. pneumoniae*, a Gram-positive pathogen for which the activity of benzodioxane–benzamides was not reported before. Thus, these compounds proved active against bacteria with different morphologies (ovococcal *S. aureus* and *S. pneumoniae*, rod-like *B. subtilis*, and *E. coli*) within the Gram-positive and Gram-negative types, as reasonably expected for inhibitors acting on the widespread FtsZ.

The FZ derivatives tested in this work demonstrated a bactericidal effect, following a mechanism of action that aligns perfectly with the current understanding of the role of FtsZ in *S. pneumoniae*. In particular, the inhibition of FtsZ by the benzodioxane–benzamides had devastating effects not only on the cell division process but also on cell elongation, resulting in a simultaneous block of the two processes [47,48].

Delving deeper into the data, the antibacterial activity indicates that the potency of the benzodioxane–benzamides against *S. pneumoniae* is closely related to the length of the linker (see Table 1). Specifically, FZ21S, FZ73, FZ94, and FZ101, which have a methylenoxy linker between the benzodioxane moiety and the benzamide scaffold, were found to be completely inactive. A difference in activity, with a preference for compounds containing an ethylenoxy chain, was also observed in *S. aureus*. Notably, FZ94 and FZ101, which feature a methylenoxy linker, showed great promise, although they were slightly less potent than FZ95 and FZ100, their superior homologs, respectively [33]. In *S. aureus*, our in-house docking studies suggested that derivatives presenting an ethylenoxy spacer linker should maintain the same binding mode as compounds with a methylenoxy linker and have a better docking score. Although a crystal structure for *S. pneumoniae* FtsZ is not yet available, it seems reasonable to hypothesize that the benzodioxane–benzamides bind FtsZ in a similar conserved sequence. Therefore, it is likely that the binding pocket could have similar characteristics across different bacterial species.

Moreover, among the compounds having a functionalization on the ethylenoxy linker (FZ116, FZ117, FZ118, and FZ119), a complete difference in activity could be observed: the *erythro* isomers, FZ116 and FZ118, are more potent than the corresponding *threo* ones, FZ117 and FZ119, which resulted in being completely inactive for this species. Such a difference for FZ116 and FZ117 was previously noticed in *E. coli* [39], *S. aureus*, and *B. subtilis* [36].

These differences lead us to speculate that the binding with the protein is, in some way, influenced by the spatial orientation of the linker substituent and the nature of the pendant group. Indeed, the chemical nature clearly affects the antibacterial potency, as FZ116, which has a hydroxyl group, is more potent than FZ118, which has a methyl group.

What is even more interesting for *S. pneumoniae* is that the chemical nature of the pendant group shows a strong impact on pneumococcal cells (see Figure 3). The presence of the hydroxyl group as a pendant, which certainly results in a more polar compound, shifts the antibacterial effect from being merely bactericidal (as observed with FZ95, FZ100, and FZ118) to also being rapidly bacteriolytic. The lytic potency of FZ116, which was able to induce morphological changes already after 15 min from the addition of the compound, suggests that the OH group might also affect other cellular processes. We will certainly study this feature in greater detail to gain further insights into possible additional effects or interactions. Nevertheless, images of the *S. pneumoniae* cells treated with any of the four benzodioxane–benzamides show morphological alterations consistent with the concomitant block of sidewall elongation and cell division, which is the characteristic phenotype of FtsZ inactivation in this species.

Finally, in vitro analysis in crowding cytomimetic conditions revealed important alterations in the polymerization features of FtsZ caused by the compounds identified as active in in vivo tests. This is consistent with benzodioxane–benzamides targeting FtsZ in *S. pneumoniae*. Interestingly, the investigated compounds hyperstabilize FtsZ polymers to varying degrees, similar to what has been previously described for *E. coli* FtsZ [39]. This suggests that the mechanisms of action in these two species may share certain similarities. Furthermore, compounds FZ95 and FZ100, along with FZ116, for which significant activity was found here on *S. pneumoniae*, are also the most active in *E. coli* FtsZ, further supporting similarities in the mode of action of the compounds in these bacteria.

## 4. Materials and Methods

### 4.1. Antimicrobial Activity on S. pneumoniae

*S. pneumoniae* strains used in this study were the well-characterized Rx1 strain and the unencapsulated laboratory derivative (Δcps) of the serotype 2 D39 strain, progenitor of Rx1 [48]. Strains were routinely grown in Tryptone Soya agar (TSA; Oxoid) supplemented with 5% defibrinated sheep blood (or Tryptone soya blood agar [TSBA]) or Tryptone soya broth (TSB; Oxoid, Hampshire, UK) at 37 °C statically.

MICs were determined by a dilution assay on plates. Tryptic Soy Agar + 5% blood (TSBA) plates containing graded amounts of the specific FZ-benzamides tested were prepared and used to determine the MIC for each compound by spotting 10 µL of an inoculum of 5x10^6^ CFU/mL of *S. pneumoniae* cells; 10 µL of H_2_O was used as a negative control. A plate without an inhibitor was used as a positive control for growth.

The effect of the selected FZ-benzamides on the growth and viability of the *S. pneumoniae* Rx1 strain was evaluated, as briefly described. Cells from frozen glycerol starters (0.3 OD) were inoculated into pre-warmed Tryptic Soy Broth and grown at 37 °C to an OD of 0.2. At this point, the culture was split into 5 tubes with or without the specific inhibitor (1× MIC) and grown at 37 °C for additional 3 h. Samples for OD measurements at 650 nm (Biochrom (Biochrom Ltd., Cambridge, UK) Ultrospec™ 7000 PC UV-Vis Spectrophotometer) and samples for microscopy were taken at T0, T5, T15, and T30 and then every 30 min up to T180. Samples for viable counts were taken at T0, T90, and T180. The results are representative of at least two independent experiments.

For phase-contrast microscopy, 1 mL of culture was transferred to Eppendorf tubes, centrifuged, resuspended, and fixed in 50 to 100 of a 4% paraformaldehyde solution (Immunofix; Bio-Optica, Milano, Italy). Aliquots of 10 μL were transferred to poly-L-lysine-coated slides and examined using a Leica (Leica, Wetzlar, Germany) DMI8 microscope equipped with a 100× phase-contrast objective. Photographs were taken with an Andor Zyla 4.2 PLUS camera.

### 4.2. In Vitro Activity of Benzodioxane–Benzamides Under Crowded Conditions

*S. pneumoniae FtsZ purification and labeling*. FtsZ was purified essentially following a previously described protocol [49] based on calcium-induced precipitation of the protein. Briefly, the lysate of *E. coli* C41 cells (transformed with the plasmid pMKV18) was centrifuged and, after the addition of 2 mM GTP (Merck, Rahway, NJ, USA) and 20 mM CaCl_2_ to the supernatant and incubation (15 min, 30 °C), FtsZ polymers were collected by centrifugation. The pelleted protein was resuspended and centrifuged, and the supernatant was subjected to a second calcium/GTP cycle. The protein was further purified by ion exchange chromatography. Fractions were pooled and stored in aliquots containing 10% glycerol at −80 °C until use. Covalent labeling of FtsZ at the amino groups with Alexa Fluor 488 carboxylic acid succinimidyl ester dye (Thermo Fisher Scientific, Waltham, MA, USA) was conducted under conditions ensuring minimal interference of the dye with the protein polymerization, as reported earlier for *E. coli* FtsZ [22,50]. Briefly, FtsZ was dialyzed in 20 mM Hepes, 50 mM KCl, 5 mM MgCl_2_, and 1 mM EDTA pH 8.0, polymerized with 20 mM CaCl_2_ and 2 mM GTP and, after incubation with the dye at a 1:2 protein:dye molar ratio (15 min, 30 °C), polymers were collected by centrifugation (15 min at 50,000 rpm in a MLA-130 rotor, 4 °C). The labeled polymers were resuspended in cold 50 mM Tris-HCl, 500 mM KCl, and 5 mM MgCl_2_ pH 7.5, centrifuged as above, and the supernatant was loaded onto a HiTrap desalting column. The labeling ratio was 0.5–0.6 moles of dye per mole of protein. The labeled protein was stored in aliquots with 10% glycerol at −80 °C until use.

*Turbidity measurements.* Depolymerization profiles of 12 µM FtsZ, in 50 mM Tris-HCl, 100 mM KCl, and 5 mM MgCl_2_ pH 7.5, and including 150 g/L Ficoll 70 (GE Healthcare, Chicago, IL, USA), were monitored in a Varioskan Flash plate reader (Thermo Fisher Scientific, MA, USA) following the absorbance at 350 nm with time, using clear, flat bottom, half-area 96 well plates (Corning, New York, NY, USA). Before use, Ficoll 70 was dialyzed in 50 mM Tris-HCl pH 7.5, 100 mM KCl, and its final concentration was measured from the refractive index of the solution, as described [51]. The MgCl_2_ concentration was adjusted to be 5 mM in the samples with FtsZ, with or without the compounds (20 µM), which were added from stock solutions in DMSO, rendering a 0.5% residual percentage that did not affect the protein behavior (Figure 4a). Data acquisition started immediately after the addition of GTP to trigger polymerization, and background contribution was subtracted in all cases.

*Confocal microscopy*. Imaging experiments were performed in a Leica TCS-SP5 inverted confocal microscope equipped with an HCX PL APO 63× oil immersion objective (N.A. 1.4, Leica, Mannheim, Germany) and a 488 nm laser line used to excite Alexa Fluor 488 dye. Samples of FtsZ with or without the compounds were prepared, as described above, for turbidity measurements, and 1 µM FtsZ-Alexa 488 was included as a tracer. Solutions were placed in silicon chambers glued to a glass coverslip for visualization. Polymerization was triggered by adding GTP, either directly in the silicon chambers for polymers imaged at t = 0 or in Eppendorf tubes for polymers observed after incubation during the specified times. Several images from different observation fields of the samples were recorded. DMSO coming from the compounds, 0.5%, did not have an effect on FtsZ, neither before nor after polymerization (Figure 4b). Images were prepared with ImageJ [52], which was also used to obtain the intensity profiles using the straight-line tool of the software through the lines shown in the images.

## 5. Conclusions

With all this information in hand, we were able to confirm that FtsZ is, also in *S. pneumoniae*, the direct target of our benzodioxane–benzamides. These findings lead us to speculate that the binding sequence of the benzodioxane–benzamides in FtsZ is highly conserved across different bacterial species. Taken together, this work demonstrates that we have developed a highly promising class of broad-spectrum antibacterial agents active in bacterial cell division, which could be further explored for structural refinements and a deeper understanding of the mechanism of action.

## Figures and Tables

**Table 1:** Biological evaluation of the antimicrobial activity on *S. pneumoniae* of all the derivatives present in this work (* FZ21S cytotoxicity is expressed as dose (µg/mL) reducing the viability by 50% (TD50)).

*Compound*	*S. pneumoniae MICs* (μg/mL)	*MRC-5* *TD90* (μg/mL)
*FZ21S*	>100	600 * [30]
*FZ73*	>100	-
*FZ94*	>100	>800 [35]
*FZ95*	25 < MIC < 30	90 [35]
*FZ100*	25 < MIC < 30	75 [35]
*FZ101*	>100	800 [35]
*FZ116*	D39: 60 < MIC < 70 Rx1: 75 < MIC < 80	190 [36]
*FZ117*	>100	190 [36]
*FZ118*	70 < MIC < 75	190 [36]
*FZ119*	>100	100 [36]

## Data Availability

The original contributions presented in this study are included in the article. Further inquiries can be directed to the corresponding authors.

## Supplementary Materials

The following supporting information can be downloaded at https://www.mdpi.com/article/10.3390/ijms26020714/s1.
