# Supplementary material for: Targeting Bacterial Cell Division with Benzodioxane–Benzamide FtsZ Inhibitors as a Novel Strategy to Fight Gram-Positive Ovococcal Pathogens
Source: Int J Mol Sci. 2025 Jan 16;26(2):714. doi: 10.3390/ijms26020714 (PMC11765573; doi:10.3390/ijms26020714)

Targeting Bacterial Cell Division with Benzodioxane-Benzamide FtsZ Inhibitors as a Novel Strategy to Fight Gram-Positive Ovococcal Pathogens

Berenice Furlan, Marta Sobrinos-Sanguino, Marcella Sammartino, Begoña Monterroso, Silvia Zorrilla, Alessia Lanzini, Lorenzo Suigo, Ermanno Valoti, Orietta Massidda* and Valentina Straniero*

Summary

Material and methods: 3
NMR 3
HPLC 3
FZ21S 4
^{1}H-NMR 4
^{13}C-NMR 4
HPLC 5
FZ73 6
^{1}H-NMR 6
^{13}C-NMR 6
HPLC 7
FZ94 8
^{1}H-NMR 8
^{13}C-NMR 8
HPLC 9
FZ95 10
^{1}H-NMR 10
^{13}C-NMR 10
HPLC 11
FZ100 12
^{1}H-NMR 12
^{13}C-NMR 12
HPLC 13
FZ101 14
^{1}H-NMR 14
^{13}C-NMR 14
HPLC 15
FZ116 16
^{1}H-NMR 16

^{13}C-NMR 16
HPLC 17
FZ117 18
^{1}H-NMR 18
^{13}C-NMR 18
HPLC 19
FZ118 20
^{1}H-NMR 20
^{13}C-NMR 20
HPLC 21
FZ119 22
^{1}H-NMR 22
^{13}C-NMR 22
HPLC 23

Material and methods:

NMR: Final compounds were dissolved in DMSO-d6 and analysed by using Mercury 300 NMR spectrometer/Oxford Narrow Bore superconducting magnet operating at 300 MHz for all ^{1}H-NMR spectra and at 75 MHz for all ^{13}C-NMR.

HPLC analyses were acquired by using reverse-phase Waters XBridge C-18 column (5 μm, 4.6 mm × 150 mm) on an Elite LaChrom HPLC system with a diode array detector (Hitachi, San Jose, CA; USA).
Mobile phase: A, H_2O + TFA 0.1%; B, acetonitrile +TFA 0.1%, linear gradient from 90% A to 10% A in 20 minutes and isocratic 90% B for additional 5 minutes; 1 mL/min as flow rate.
The purity of the compounds was quantified at 280 nm, the peculiar λ max value, and all resulted in >95%. The relative retention times and area % are reported in each spectrum.

FZ21S

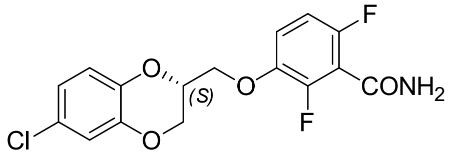

FZ21S

^{1}H-NMR

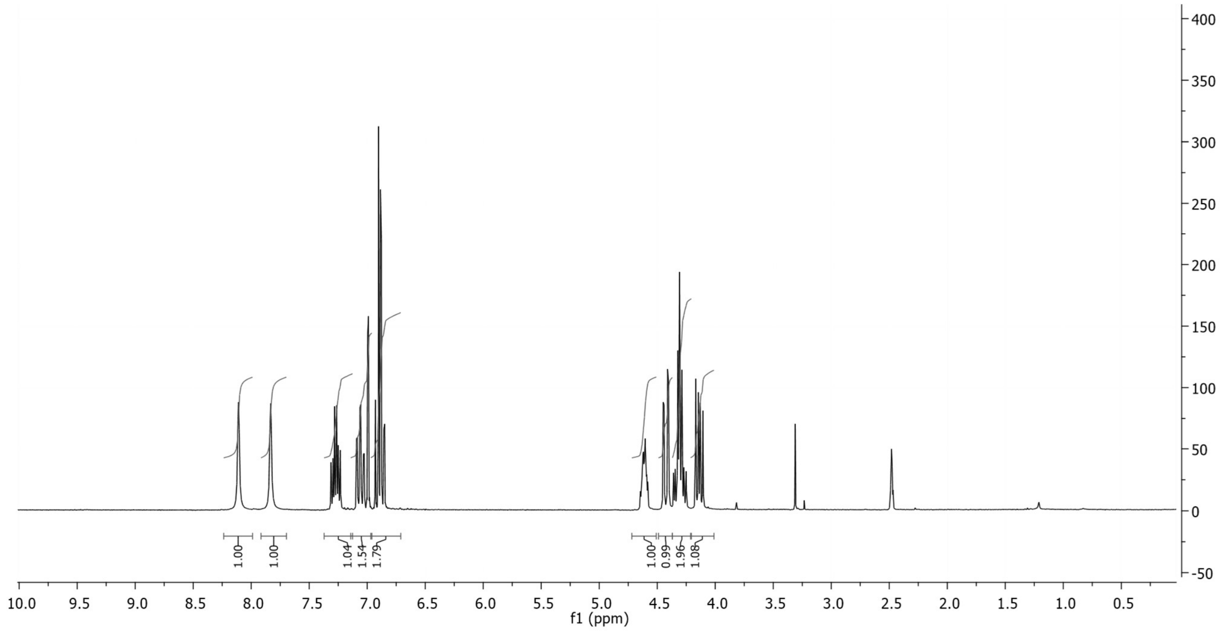

^{13}C-NMR

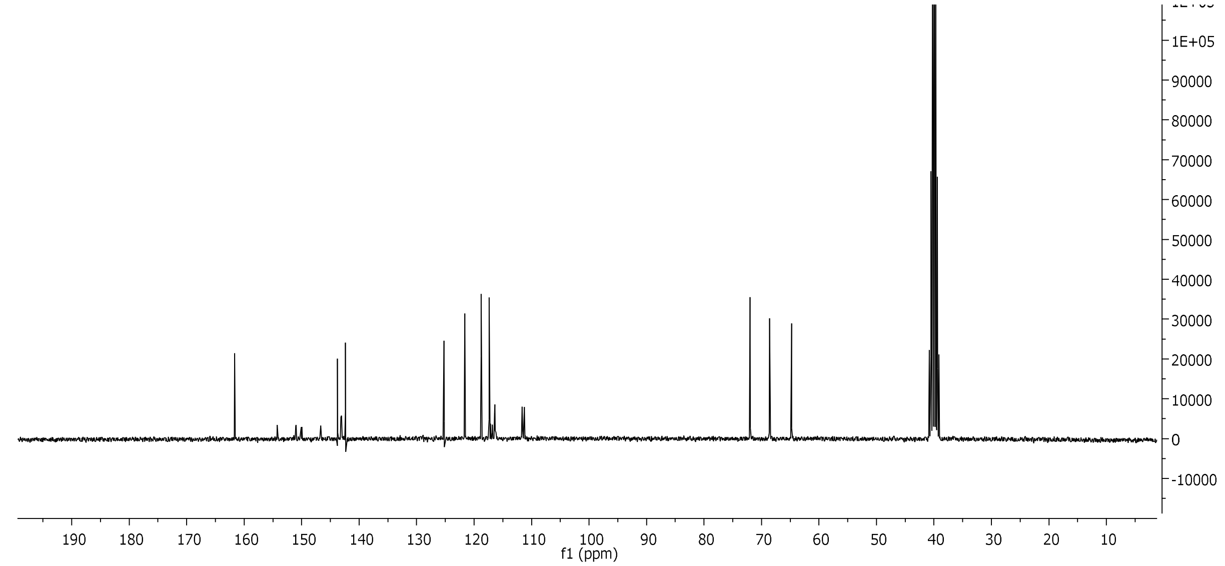

HPLC

Rt: 18.2 min, A%: 99.6%

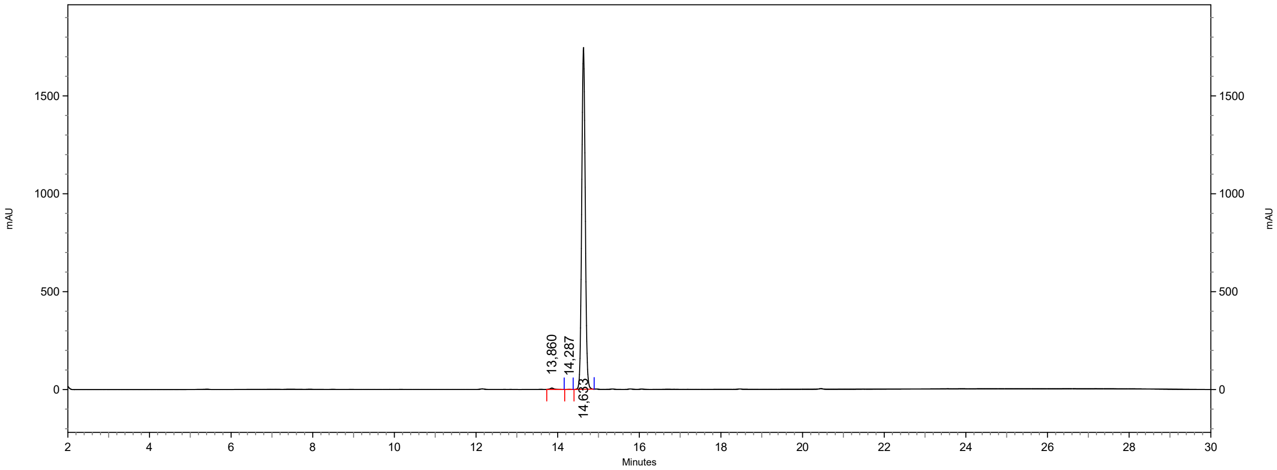

FZ73

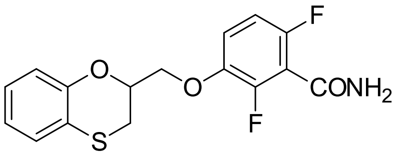

FZ73

^{1}H-NMR

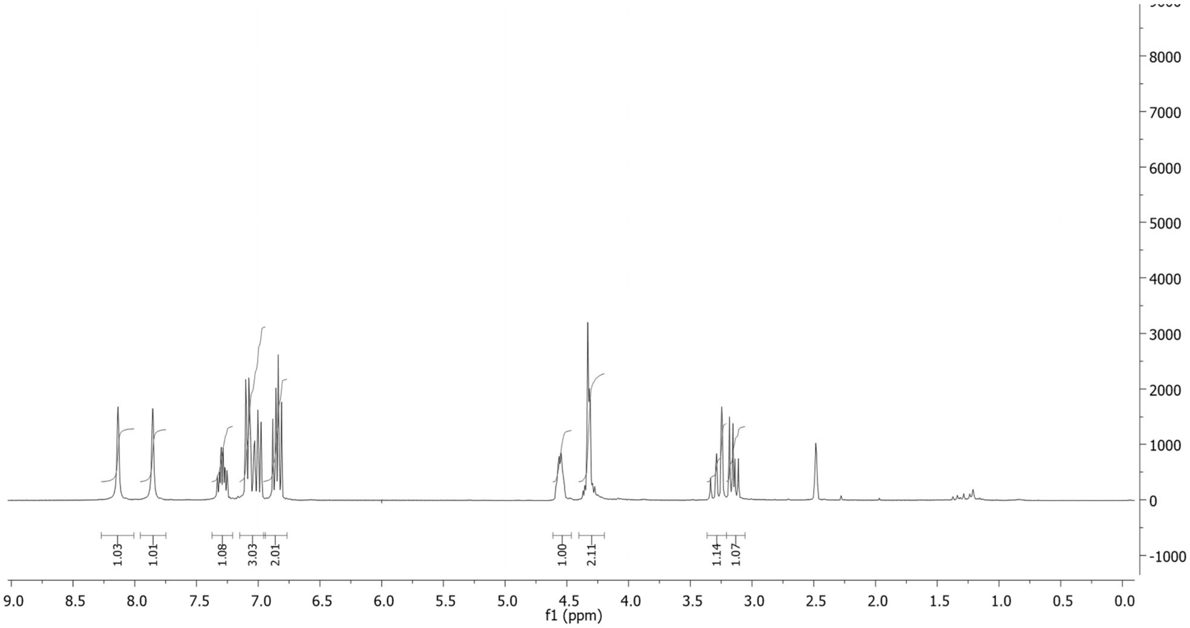

^{13}C-NMR

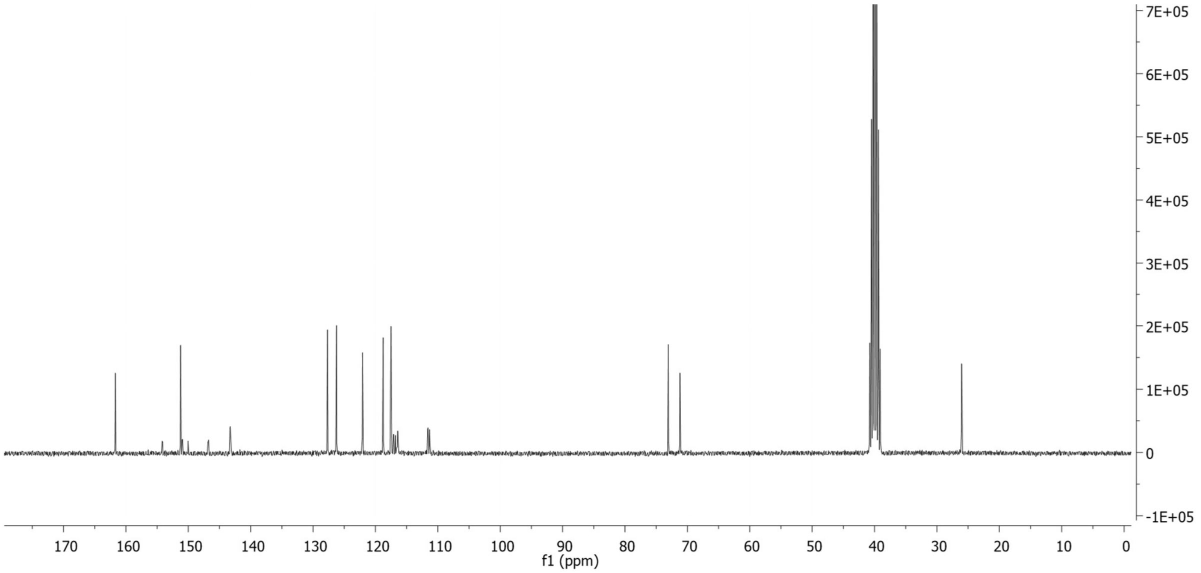

HPLC

Rt: 13.5 min, A%: 97.2%

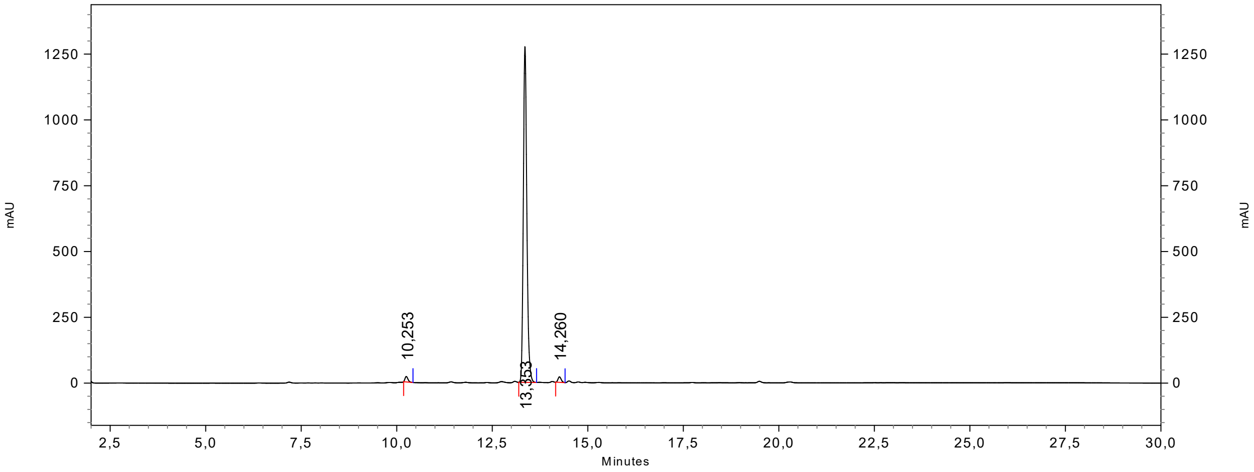

FZ94

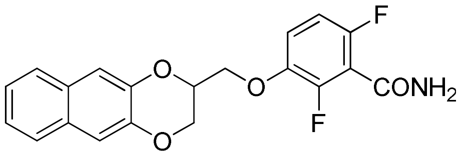

FZ94

^{1}H-NMR

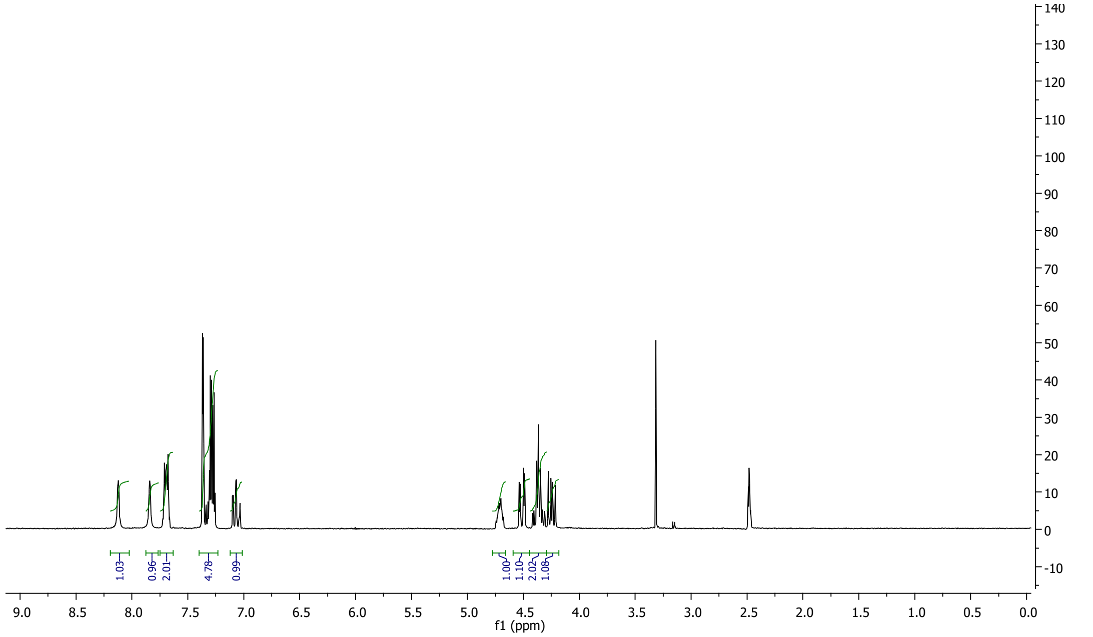

^{13}C-NMR

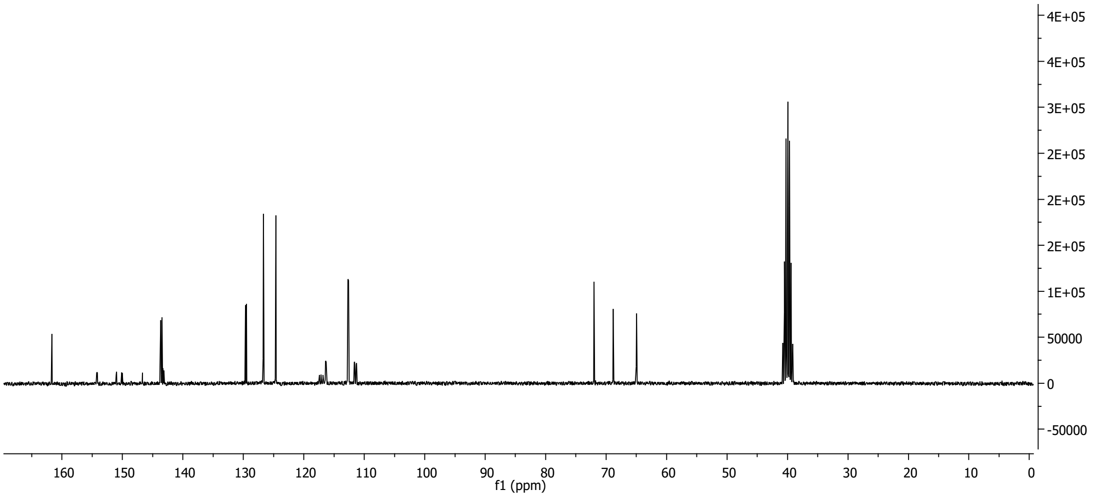

HPLC

Rt: 14.5 min, A%: 96.8%

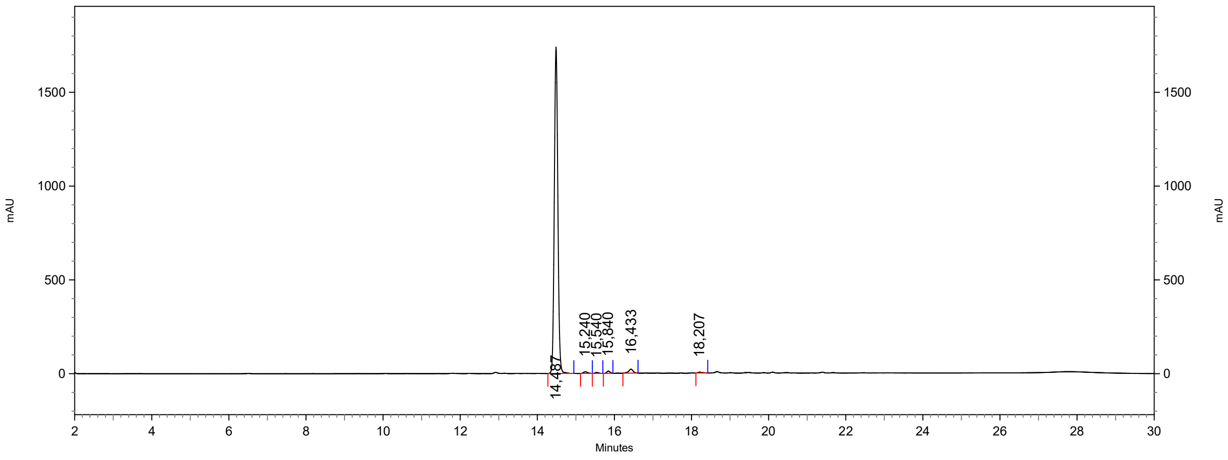

FZ95

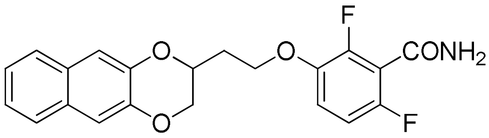

FZ95

^{1}H-NMR

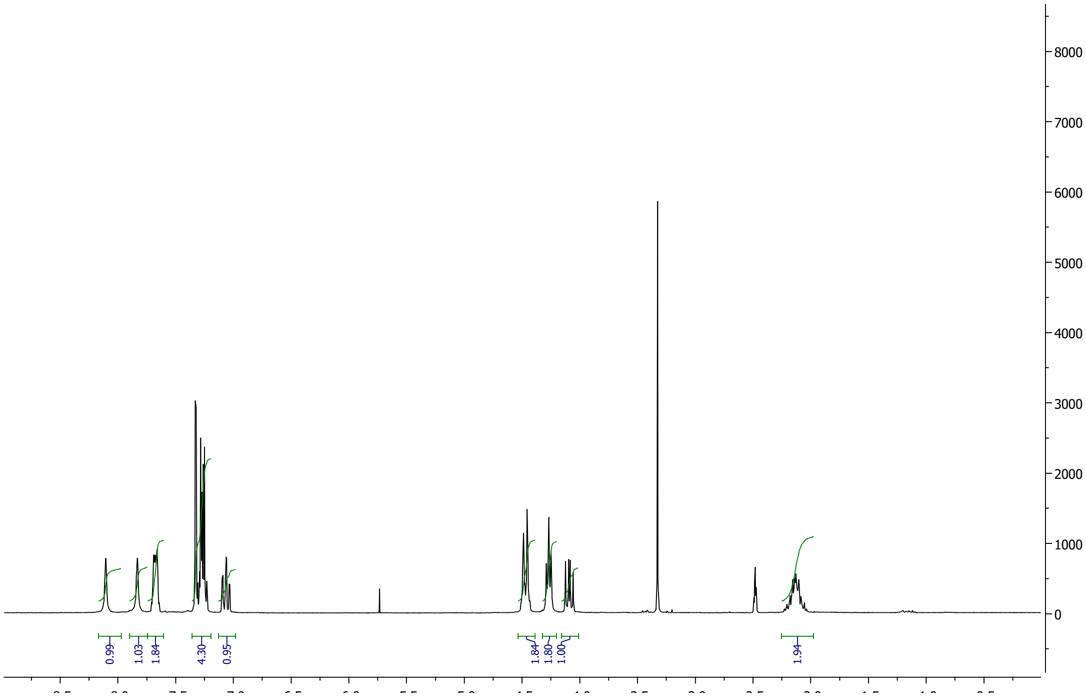

^{13}C-NMR

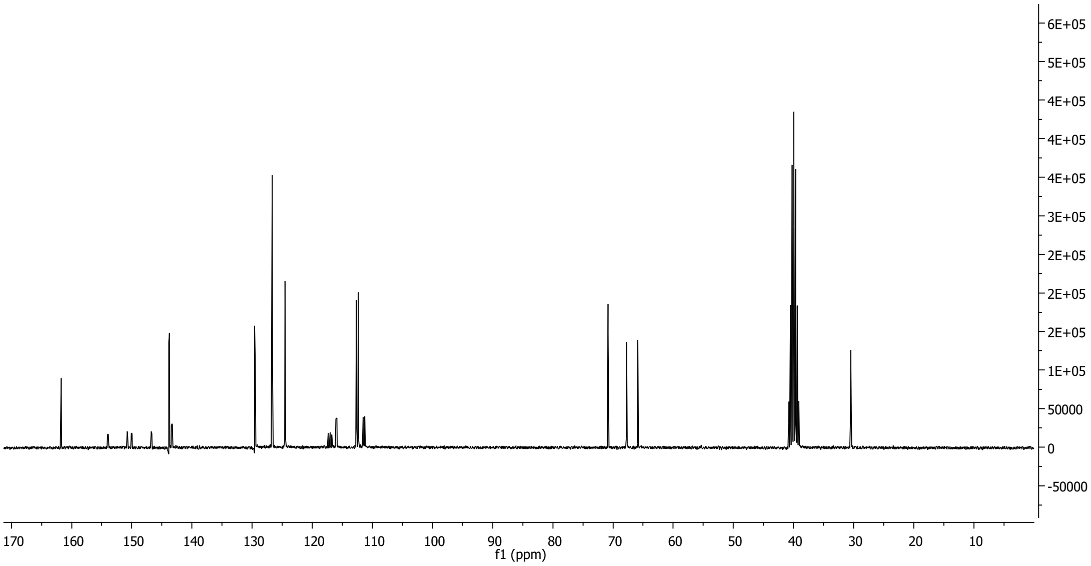

HPLC

Rt: 15.2 min, A%: 98.6%

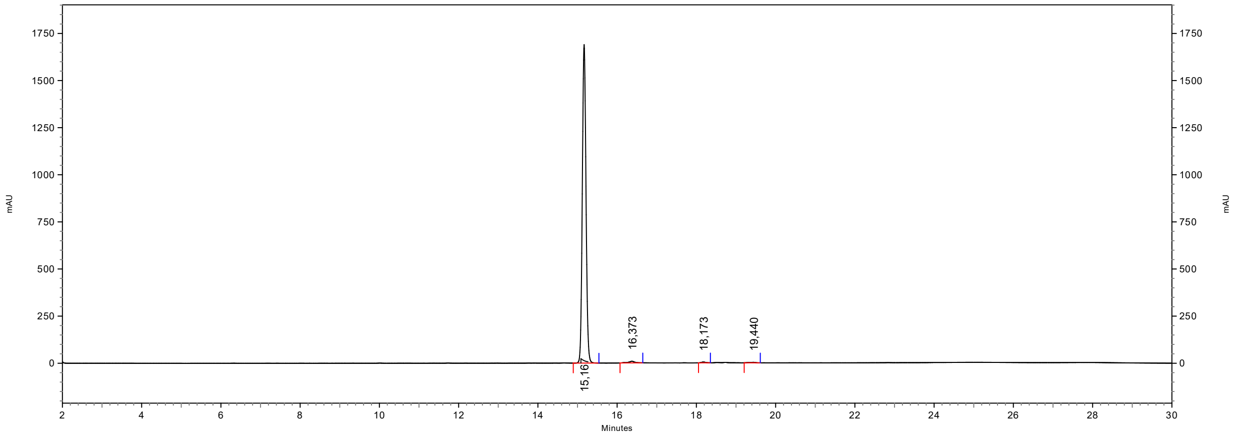

FZ100

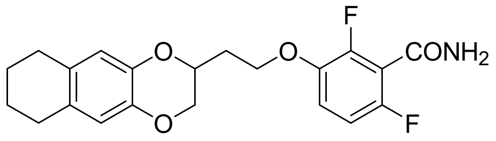

FZ100

^{1}H-NMR

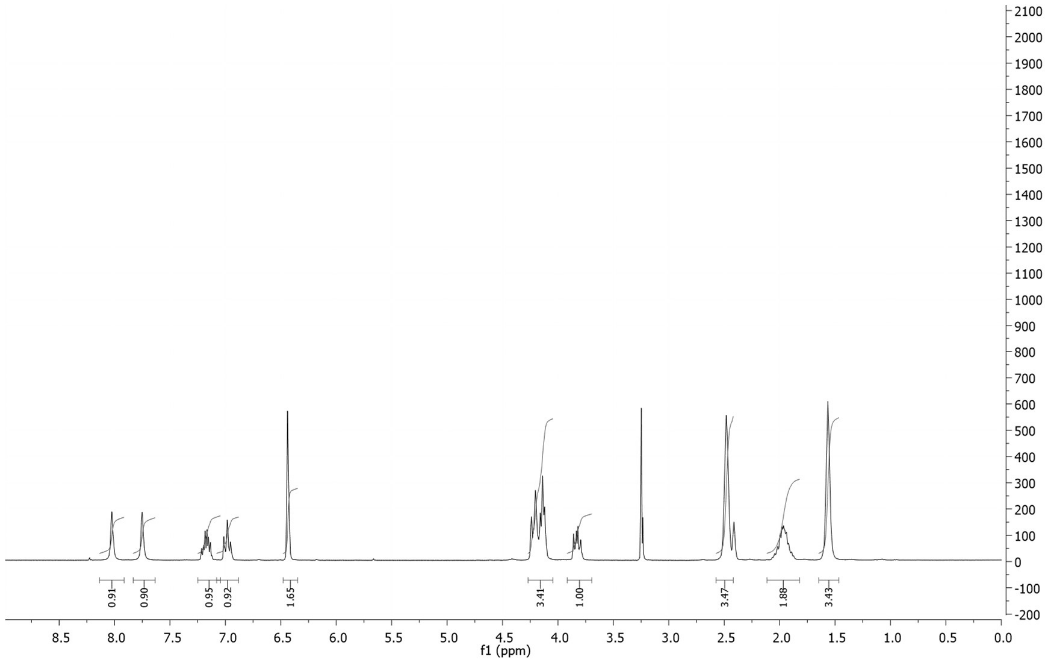

^{13}C-NMR

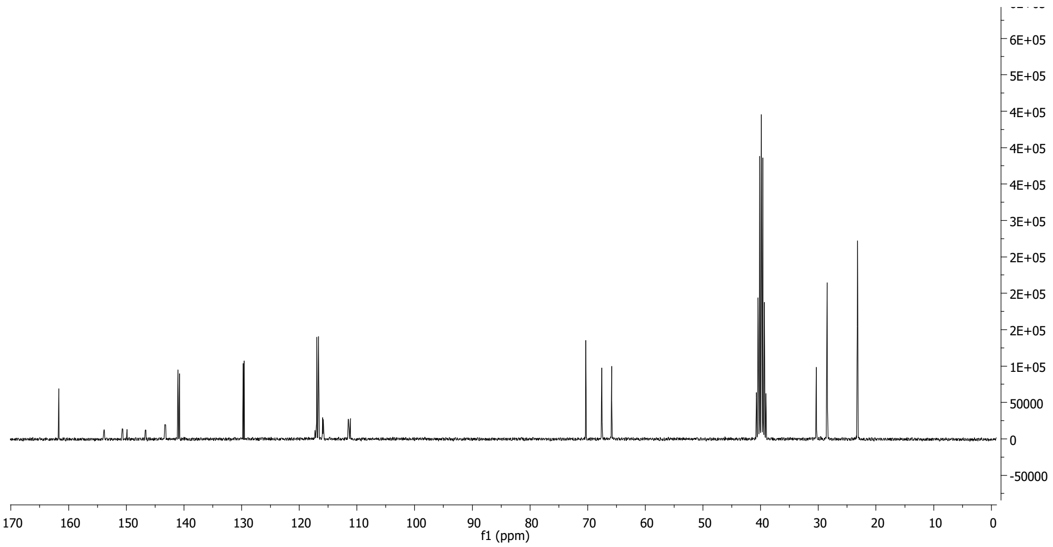

HPLC

Rt: 16.7 min, A%: 95.2%

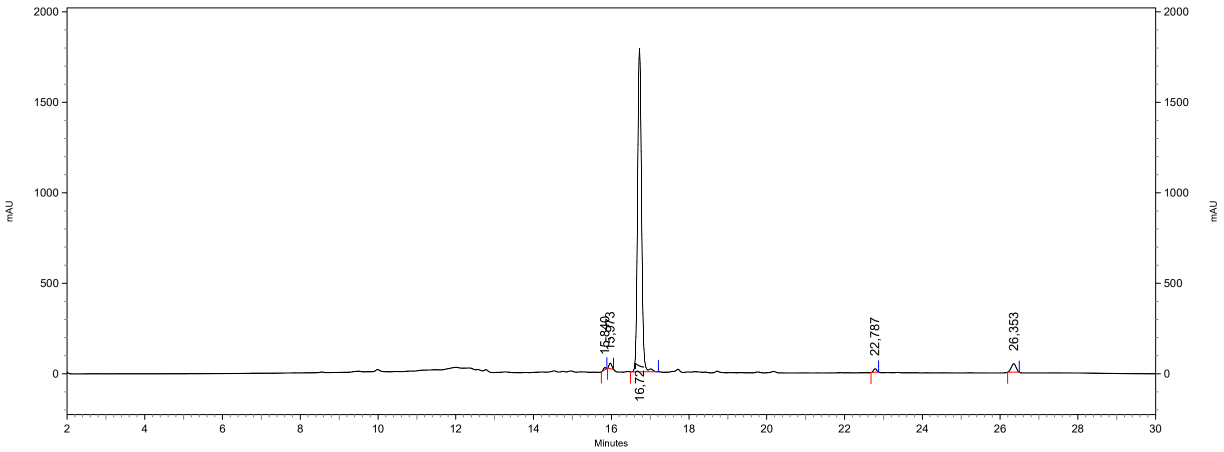

FZ101

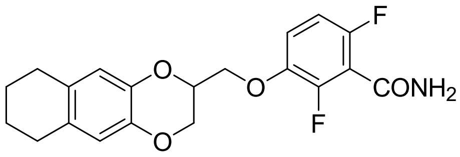

FZ101

^{1}H-NMR

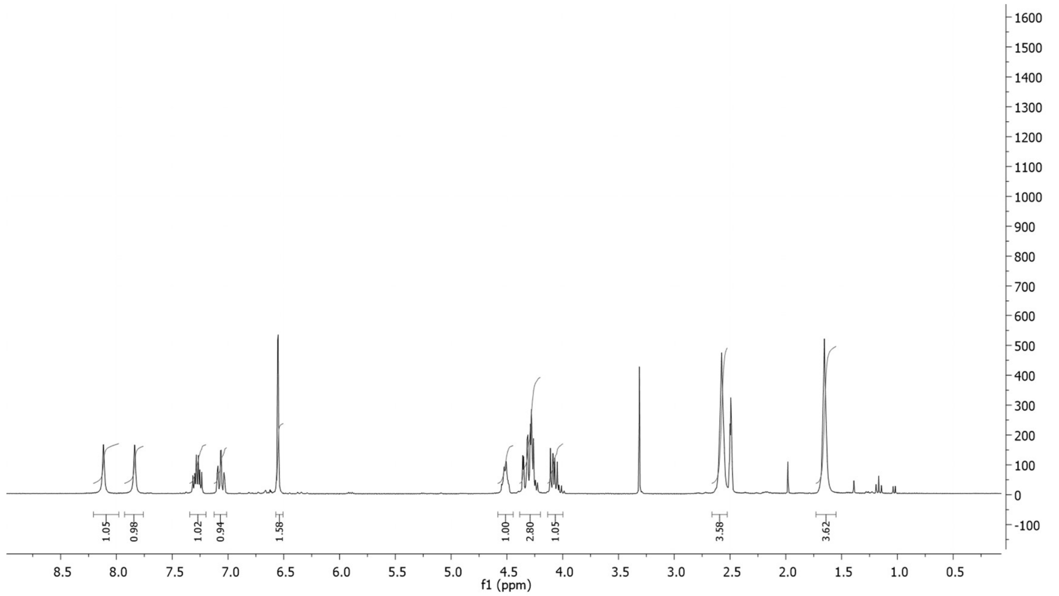

^{13}C-NMR

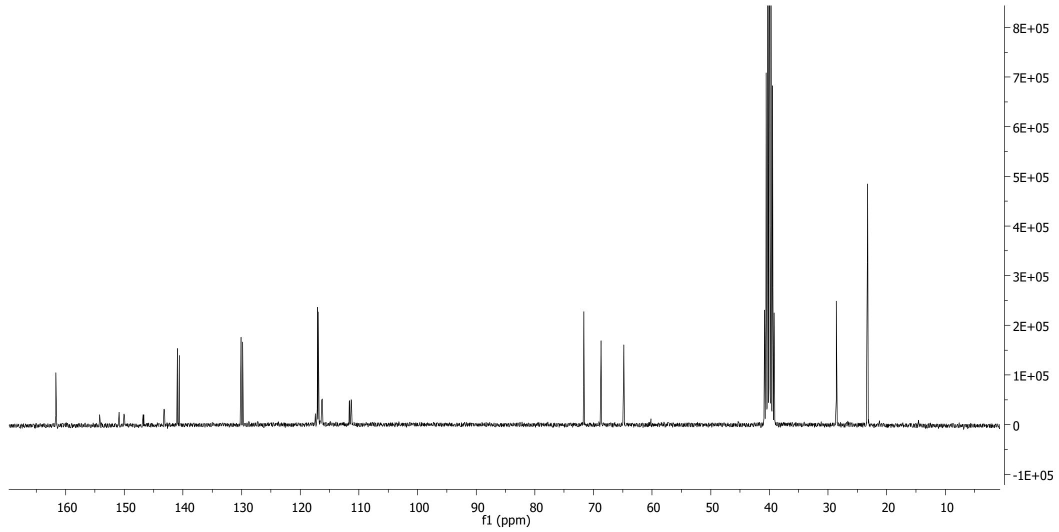

HPLC

Rt: 16.6 min, A%: 95.5%

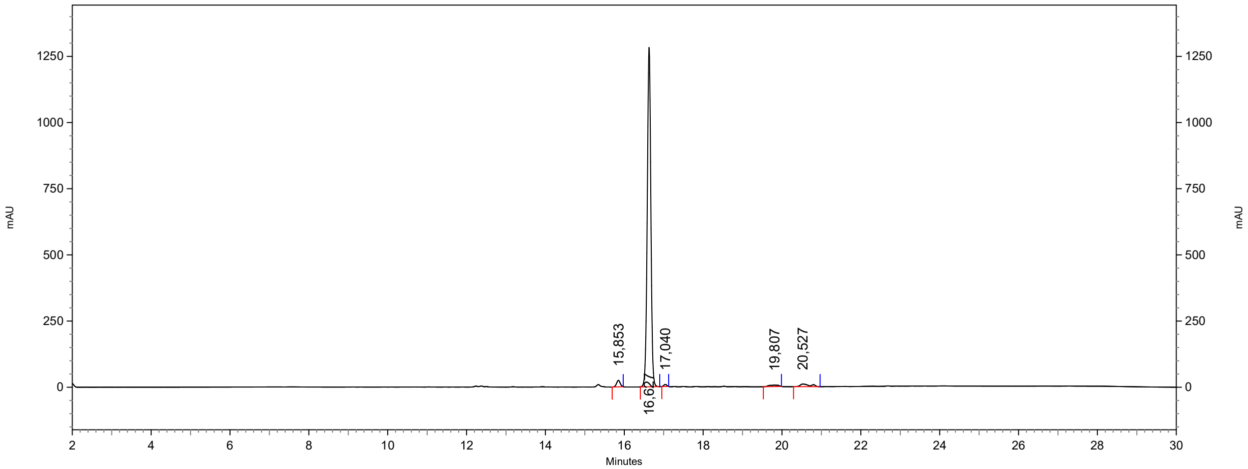

FZ116

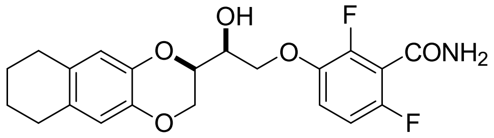

FZ116 (*Erythro*)

^{1}H-NMR

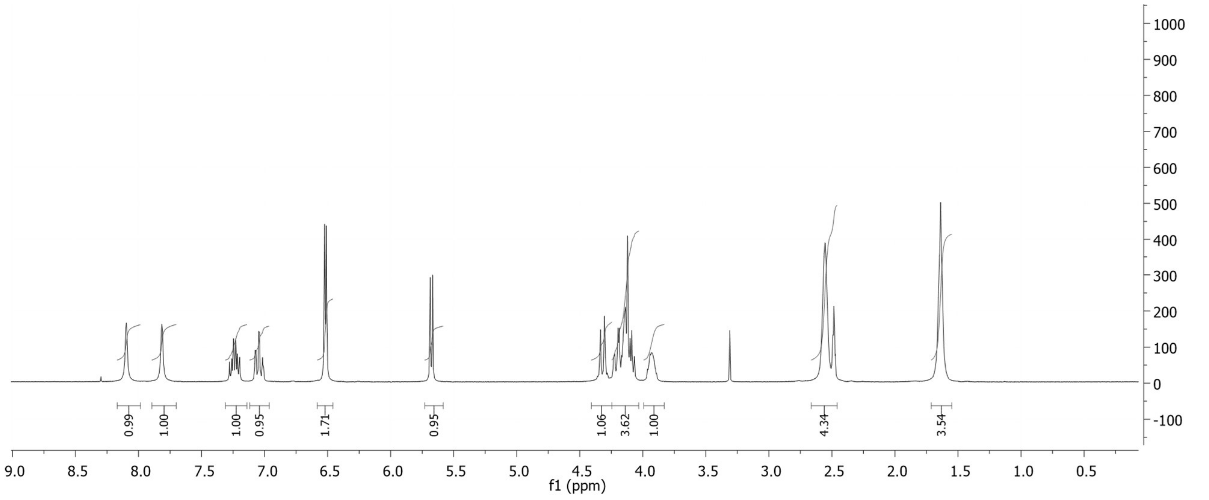

^{13}C-NMR

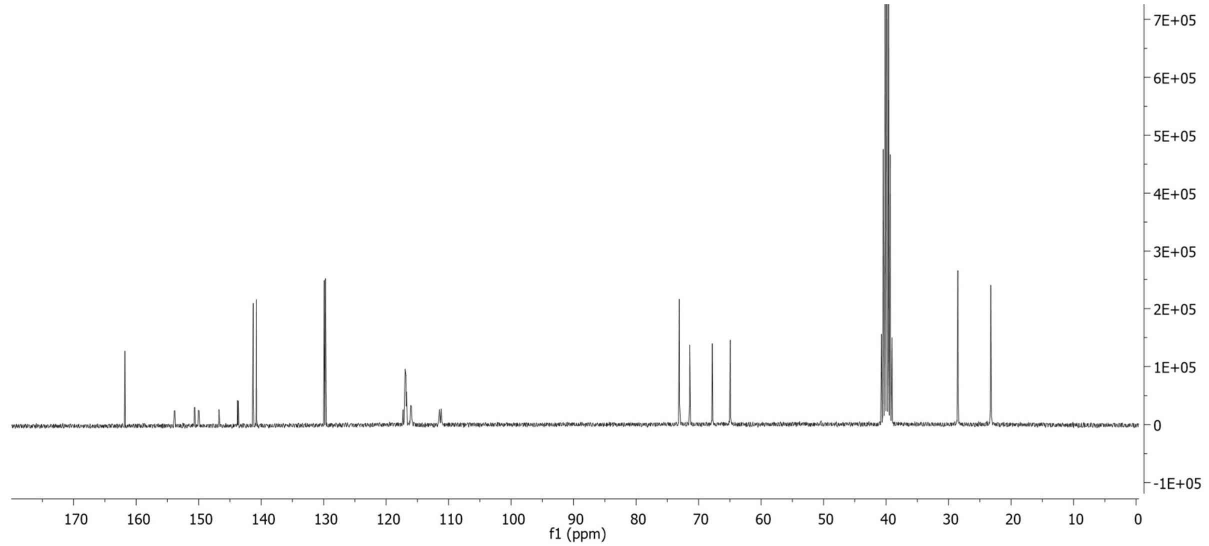

HPLC

Rt: 15.0 min, A%: 99.9%

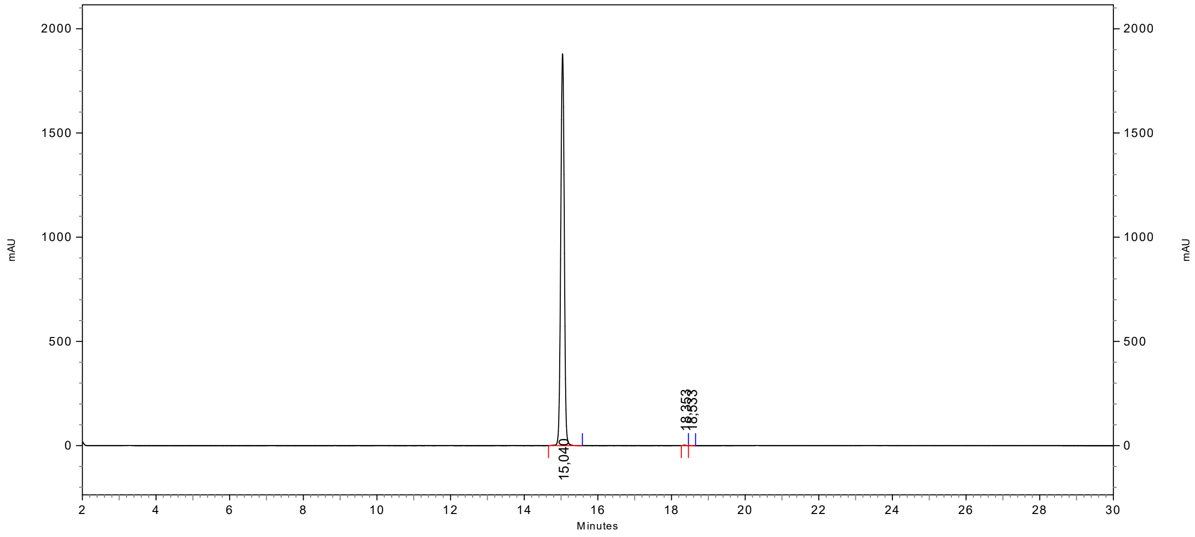

FZ117

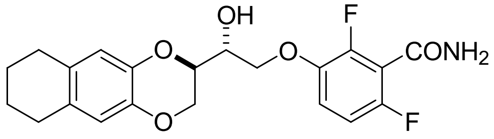

FZ117 (*Threo*)

^{1}H-NMR

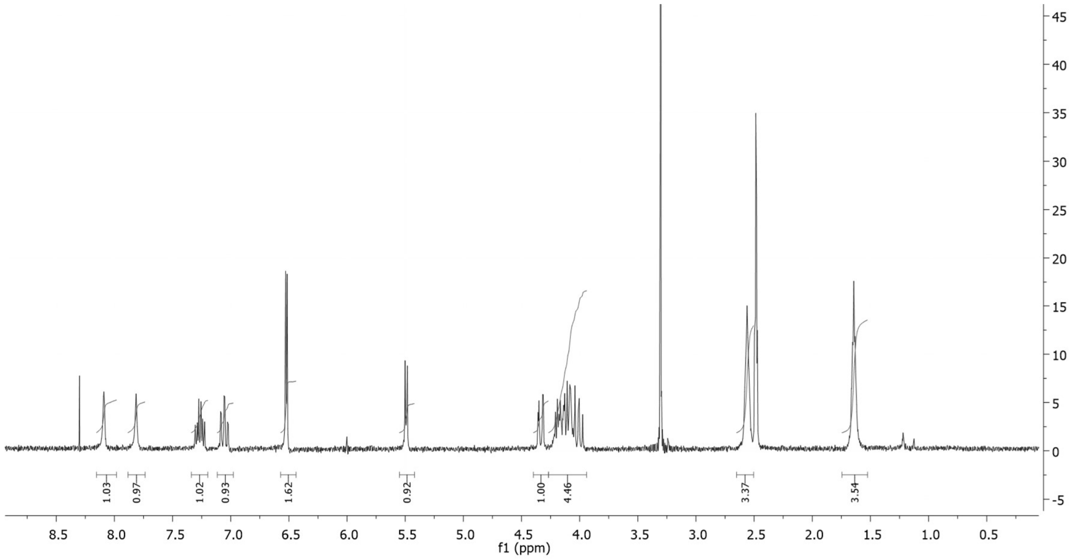

^{13}C-NMR

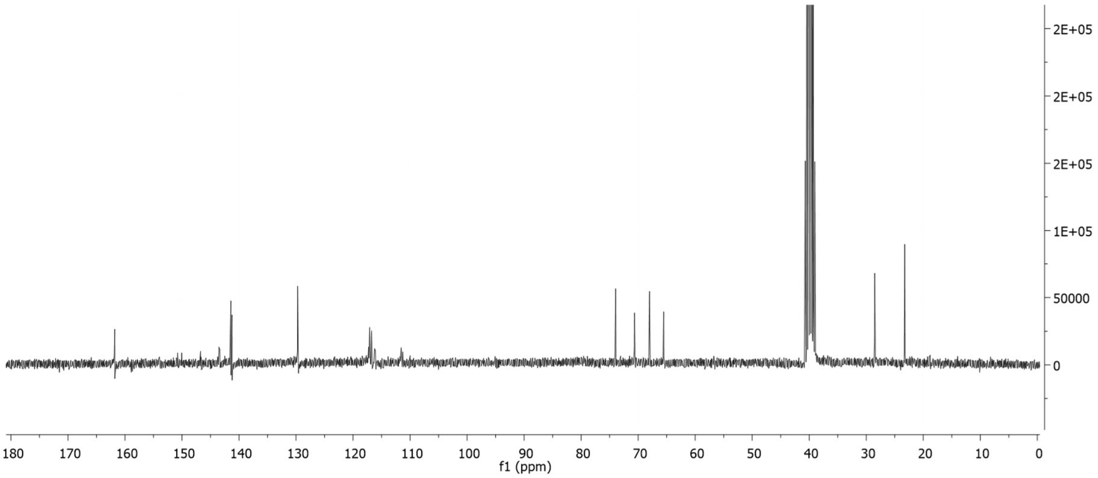

HPLC

Rt: 14.6 min, A%: 99.9%

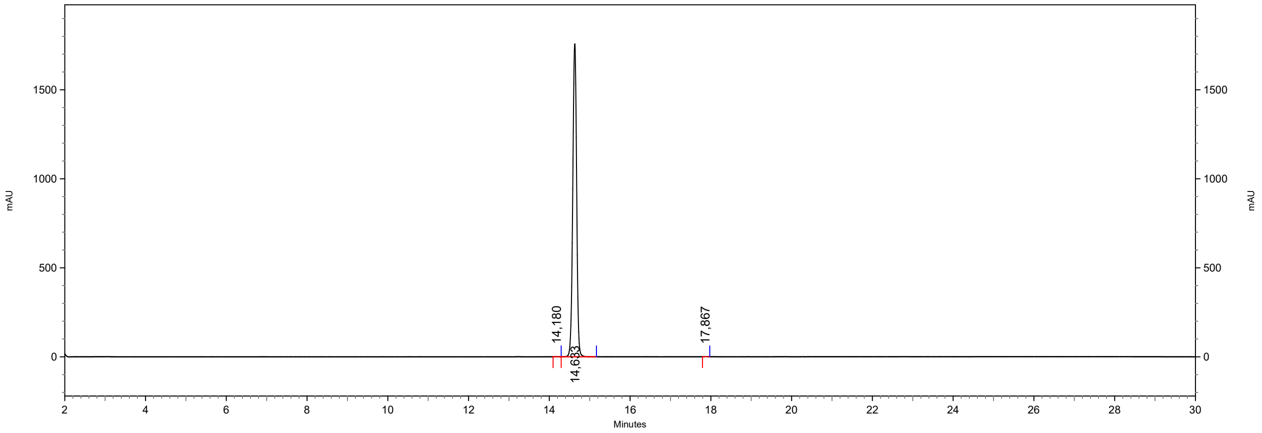

FZ118

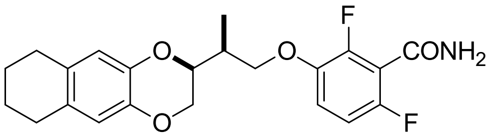

FZ118 (*Erythro*)

^{1}H-NMR

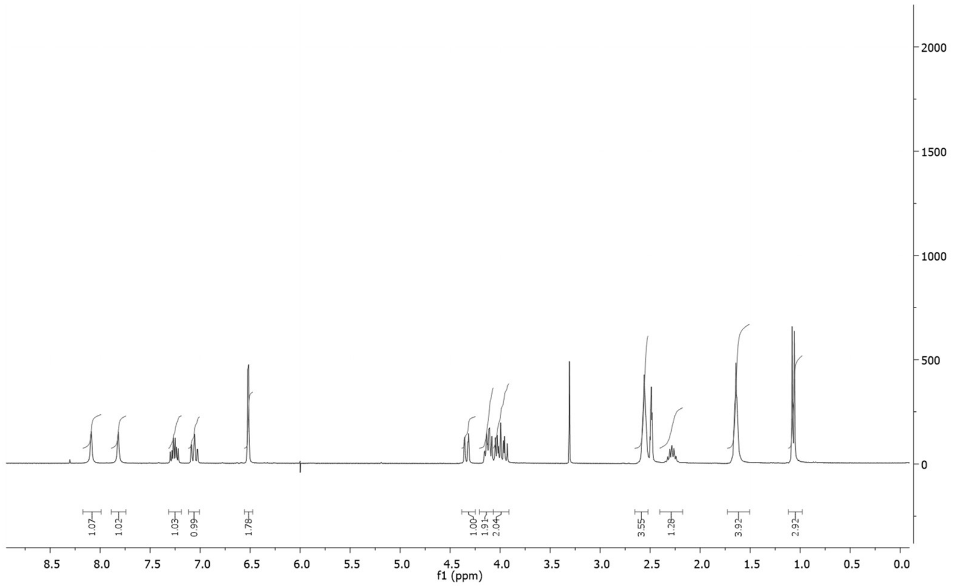

^{13}C-NMR

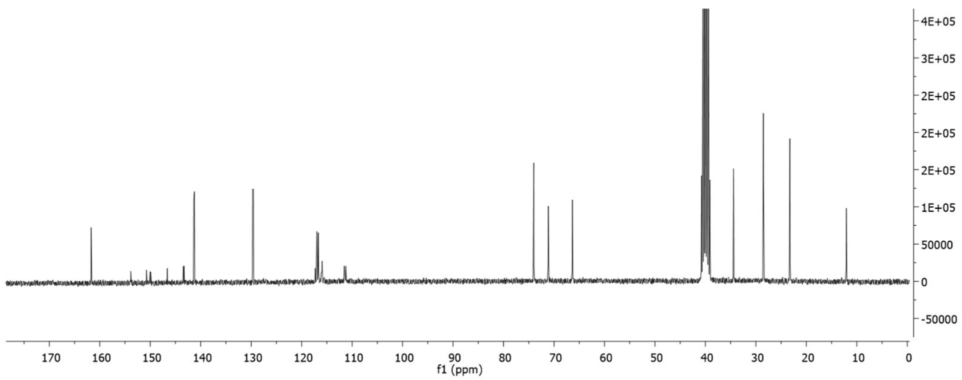

HPLC

Rt: 18.2 min, A%: 96.5%

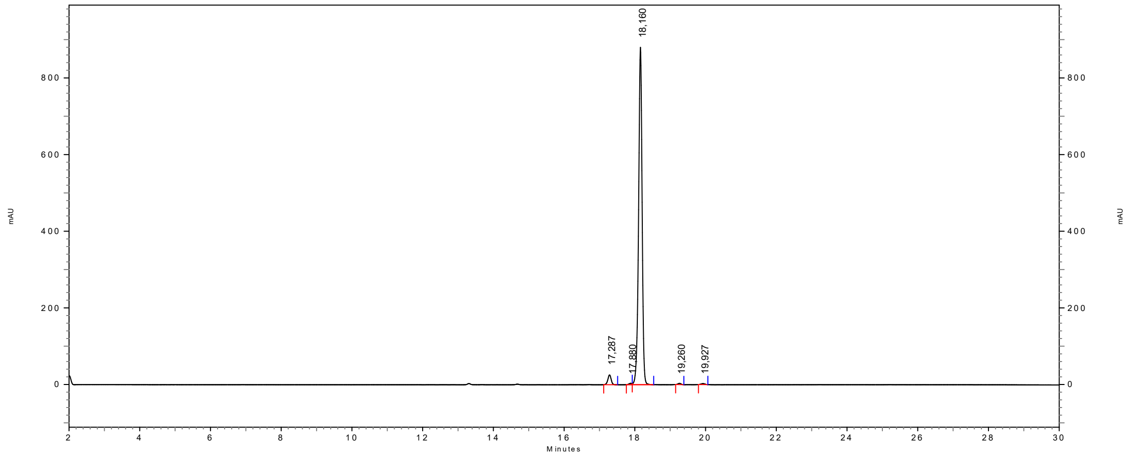

FZ119

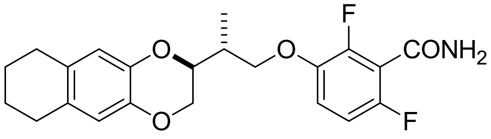

FZ119 (*Threo*)

^{1}H-NMR

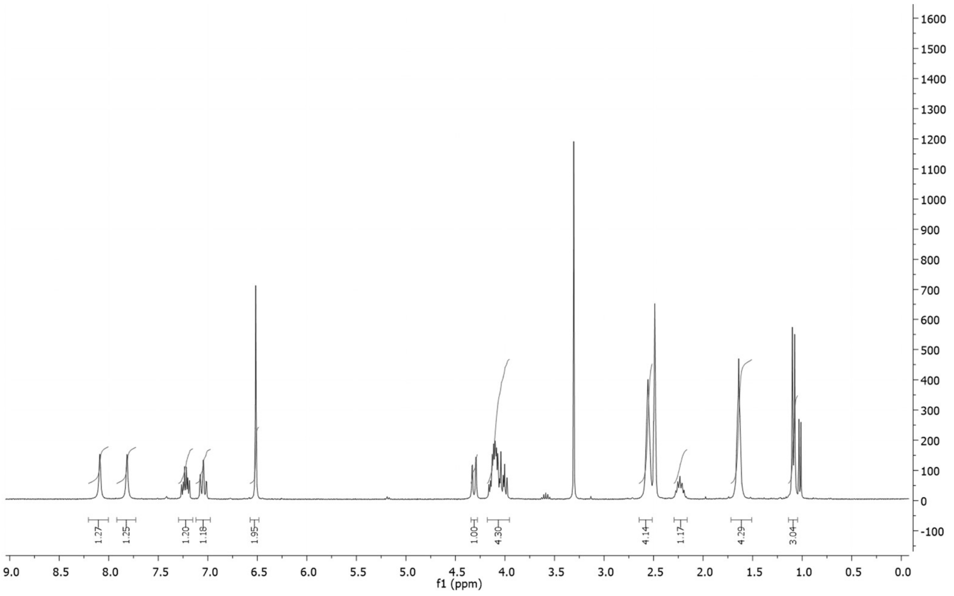

^{13}C-NMR

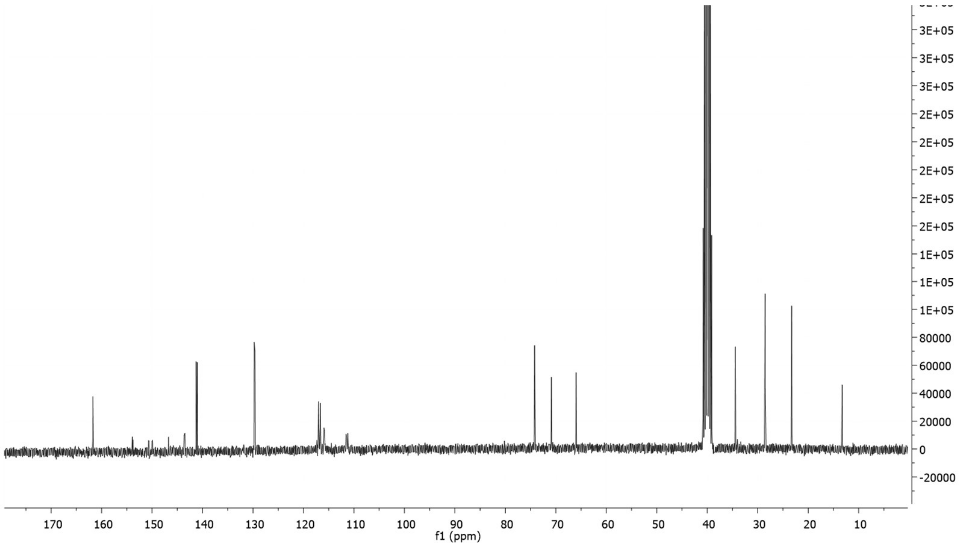

HPLC

Rt: 17.8 min, A%: 96.0%

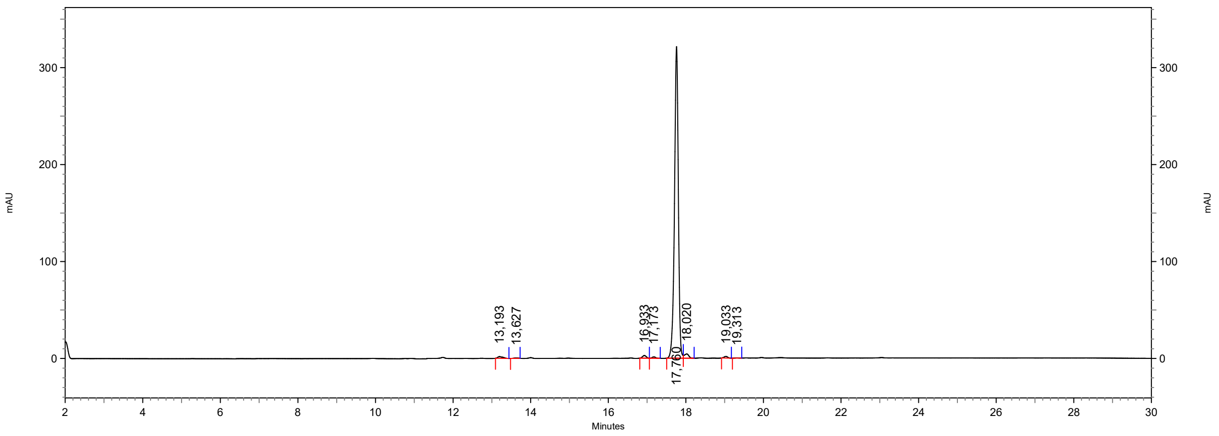

Supplement: Supplementary file 1 [file ijms-26-00714-s001.zip › ijms-3395918-supplementary.pdf]
